# Sm2, a paralog of the *Trichoderma* cerato-platanin elicitor Sm1, is also highly important for plant protection conferred by the fungal-root interaction of *Trichoderma* with maize

**DOI:** 10.1186/s12866-014-0333-0

**Published:** 2015-01-16

**Authors:** Romana Gaderer, Netta L Lamdan, Alexa Frischmann, Michael Sulyok, Rudolf Krska, Benjamin A Horwitz, Verena Seidl-Seiboth

**Affiliations:** Research Division Biotechnology and Microbiology, Institute of Chemical Engineering, Vienna University of Technology, Gumpendorfer Strasse 1a, 1060 Vienna, Austria; Department for Agrobiotechnology (IFA-Tulln), University of Natural Resources and Life Sciences (BOKU), Vienna, Austria; Department of Biology, Technion – Israel Institute of Technology, Haifa, Israel

**Keywords:** Cerato-platanin protein, *Trichoderma virens*, *Trichoderma atroviride*, Mycoparasitism, Biocontrol, Plant protection, Maize, *Cochliobolus heterostrophus*

## Abstract

**Background:**

The proteins Sm1 and Sm2 from the biocontrol fungus *Trichoderma virens* belong to the cerato-platanin protein family. Members of this family are small, secreted proteins that are abundantly produced by filamentous fungi with all types of life-styles. Some species of the fungal genus *Trichoderma* are considered as biocontrol fungi because they are mycoparasites and are also able to directly interact with plants, thereby stimulating plant defense responses. It was previously shown that the cerato-platanin protein Sm1 from *T. virens* - and to a lesser extent its homologue Epl1 from *Trichoderma atroviride* - induce plant defense responses. The plant protection potential of other members of the cerato-platanin protein family in *Trichoderma*, however, has not yet been investigated.

**Results:**

In order to analyze the function of the cerato-platanin protein Sm2, *sm1* and *sm2* knockout strains were generated and characterized. The effect of the lack of Sm1 and Sm2 in *T. virens* on inducing systemic resistance in maize seedlings, challenged with the plant pathogen *Cochliobolus heterostrophus*, was tested. These plant experiments were also performed with *T. atroviride epl1* and *epl2* knockout strains. In our plant-pathogen system *T. virens* was a more effective plant protectant than *T. atroviride* and the results with both *Trichoderma* species showed concordantly that the level of plant protection was more strongly reduced in plants treated with the *sm2/epl2* knockout strains than with *sm1/epl1* knockout strains.

**Conclusions:**

Although the cerato-platanin genes *sm1/epl1* are more abundantly expressed than *sm2/epl2* during fungal growth, Sm2/Epl2 are, interestingly, more important than Sm1/Epl1 for the promotion of plant protection conferred by *Trichoderma* in the maize-*C. heterostrophus* pathosystem.

**Electronic supplementary material:**

The online version of this article (doi:10.1186/s12866-014-0333-0) contains supplementary material, which is available to authorized users.

## Background

Fungi belonging to the ascomycete genus *Trichoderma* inhabit the soil and rhizosphere, where they interact with plant roots and with other fungi. Agricultural biocontrol applications take advantage of the well-known ability of *Trichoderma* spp. to attack and destroy fungal hosts, which is called mycoparasitism. The wide host range includes soil-borne plant pathogens such as *Rhizoctonia solani* or *Pythium ultimum*, which make *Trichoderma* spp. biological plant protectants. In addition, the interaction of *Trichoderma* with roots primes the plant’s immune system for better resistance against pathogens [1-4]. Due to this induced systemic resistance, *Trichoderma* spp. are able to protect plants against some foliar pathogens, in addition to soil-borne pathogens. Plants recognize proteins secreted by the fungus, and such microbe associated (in this case fungal-associated) molecular patterns activate systemic resistance. The first such secreted protein to be studied in detail in *Trichoderma*-plant interactions was a small secreted cysteine-rich protein belonging to the cerato-platanin protein (CPP) family, named Sm1/Epl1 in *Trichoderma virens* and *Trichoderma atroviride*, respectively [5,6]. The *Trichoderma* genomes analyzed so far contain three genes encoding CPPs [7]. Gene expression was analyzed in *T. atroviride* and revealed that *epl1* is expressed during hyphal growth, *epl2* expression was only detected during spore maturation, and hardly any expression was found for *epl3* [8]. Single and double knockout strains of *epl1* and *epl2* did not reveal any phenotype related to hyphal growth or development.

*T. atroviride* and *T. virens* belong to distant clades within the genus *Trichoderma*, for which so far already more than 200 species have been described [9]. Analysis of the genomes of *T. virens* and *T. atroviride* revealed numerous differences in the genome inventory of these two species, which each have more than 2500 genes that do not occur in the other species [7]. Further, even strongly conserved genes, e.g. chitinases, have already been shown to be differentially expressed in *T. atroviride* and *T. virens* [10]. However, the biological consequences of these findings on the lifestyle of *T. atroviride* and *T. virens* have, as yet, only been partially understood. It is therefore important to note that it is not always valid to draw direct conclusions from the results in one species to the other. Rather, one can study protein families in both of them in order to better elucidate the similarities and differences between *T. atroviride* and T*. virens*.

One example of the differences between these two species are the CPP orthologues Sm1 and Epl1. *T. virens* Sm1 was shown to induce plant defense responses, but this ability is far weaker for its homologue *T. atroviride* Epl1 [11]. This was explained by different tendencies of these proteins to dimerize. Only monomers of Sm1 and Epl1 were shown to efficiently induce plant defense responses. While Epl1 is readily able to form dimers, Sm1 has a single glycosylation site that is not present in Epl1 and is predominantly found in its monomeric form, which is more effective in the induction of plant defense responses [11].

The ability of CPP family members of *Trichoderma* from other phylogenetic branches to promote systemic resistance in plants has not been studied yet. In this study, we generated knockout strains of *T. virens sm1* and *sm2* and analyzed them for developmental phenotypes and for their ability to induce resistance of maize to the Southern corn leaf blight pathogen *Cochliobolus heterostrophus*. Plant experiments were also carried out with *T. atroviride epl1* and *epl2* knockout strains [8].

## Results

### Gene expression of CPP-encoding genes in *T. virens*

**Figure 1:**
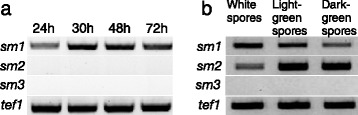
**Gene expression of** ***sm1*** **,** ***sm2*** **and** ***sm3*** **, analyzed with RT-PCR. (a)** Biomass harvested from shake flask cultivations with glucose as carbon source **(b)** Biomass harvested from sporulating fungal cultures grown on agar plates (PDA). *Tef1* was used as reference gene.

The genome of *T. virens* Gv29-8 contains three genes, *sm1*, *sm2* and *sm3*, encoding CPPs (http://genome.jgi-psf.org/TriviGv29_8_2/TriviGv29_8_2.home.html and [7]). This is in analogy to the genomes of *T. atroviride* and *T. reesei*, which also contain three CPP genes [6,8]. The genes *sm2* and *sm3* and their respective proteins have not been studied yet. In order to assess the transcriptional profiles of all three genes encoding CPPs in *T. virens*, their expression was first analyzed with RT-PCR. While the *sm1* gene was found to be expressed during hyphal growth, no expression was observed for *sm2* and *sm3* at these time points (Figure 1a). In biomass harvested from sporulating cultures grown on potato dextrose agar (PDA) plates during different stages of spore maturation, ranging from mycelium covered with white conidia to first light green and then dark green conidia, *sm2* was found to be strongly upregulated, but *sm1* was also found to be expressed (Figure 1b). For *sm3* no expression was detected under the tested growth conditions.

### Generation and characterization of *sm1* and *sm2* knockout strains

**Figure 2:**
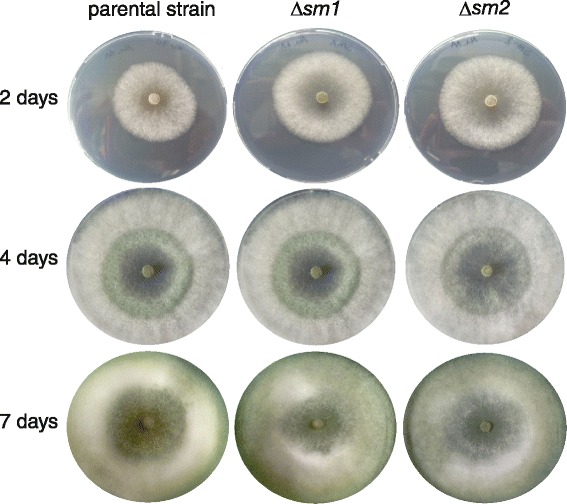
**Colony growth of** ***T. virens*** **parental strain Δ** ***tku70, sm1*** **and** ***sm2*** **knockout strains on agar plates (PDA).**

Based on these gene expression results, gene knockout strains of *sm1* and *sm2* were generated in *T. virens* I10 Δ*tku70* [12] as described in the methods section (Additional file 1: Figure S1). Since Sm1 was previously shown to be far more potent in inducing plant defense responses than Epl1 [11] and the lack of *sm1* strongly reduces the ability of *T. virens* to induce plant defense responses [13], we were interested to test whether Sm2 has a similar function in *T. virens*. Phylogenetically Sm2 belongs to a different branch of CPPs [6] and none of these proteins have been studied so far. Since *sm1* is expressed throughout different growth stages and a possible function of CPPs in fungal growth has been discussed in the literature [14], the generated knockout strains were tested for phenotypic alterations with respect to the following properties related to fungal growth: growth on agar plates (Figure 2), formation of aerial hyphae, growth along (moist) surfaces, bridging of gaps between two agar blocks, and transition of hyphae between solid/liquid interfaces. Different types of desiccation stress, e.g. drying of water droplets and drying of thin agar plates, were also examined. Furthermore, we analyzed conidiation, biomass formation in shake flask cultivations, germination efficiency, hydrophobicity of the mycelium, chlamydospore formation, osmotic stress, and cell wall stress (i.e. addition of Calcofluor white and Congo Red, both of which interfere with the construction and stress response of the cell wall [15]). No morphological differences between the parental strain and the knockout strains were found in *T. virens,* which is similar to *T. atroviride,* where no phenotype had been detected in *epl1* and *epl2* single and double knockout strains [8]. At advanced time points (48 h and 72 h) of shake flask cultivations of *T. virens* abundant formation of chlamydospores was detected (Additional file 1: Figure S2). A correlation between chlamydospore formation and *cp* (*cerato-platanin*) gene expression had been reported for *Ceratocystis platani* [16], but no differences were detected between the parental strain and the knockout strains in *T. virens*.

The mycoparasitic potential of the knockout strains was also not altered based on confrontation assays of *T. virens* against *R. solani* (Additional file 1: Figure S3) and *Botrytis cinerea* (data not shown).

**Figure 3:**
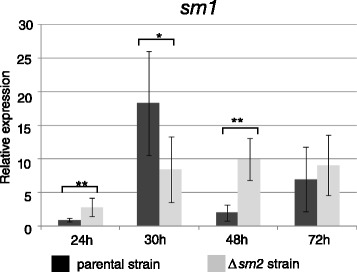
**Gene expression (qPCR) of** ***sm1*** **in the parental strain and** ***sm2*** **knockout strain.** Samples were taken at the indicated time points from shake flask cultivations with glucose as carbon source. All samples were normalized to the 24 h sample of the parental strain. *Tef1* was used as reference gene. Bars indicate the SEM (*, ** and *** indicate significance at P < 0.05, 0.01 and 0.001).

We had found in a previous study that the expression of *T. atroviride epl1,* the homologue of *T. virens sm1*, is not constant during hyphal growth but its expression level is strongly dependent on parameters that influence the growth rate (e.g. medium composition and growth temperature) [8]. We therefore paid particular attention to this aspect in the knockout strains and assessed the gene expression of *sm1* quantitatively with qPCR in the *sm2* knockout strain and the parental strain Δ*tku70* (control strain). The results (Figure 3) showed that *sm1* has a slightly different expression profile in the *sm2* knockout strain compared to the parental strain, but we were not able to elucidate this further due to the lack of any detectable morphological changes in the knockout strains.

### *T. virens* strain I10 is a Q-strain

*T. virens* strains can be grouped into P- and Q-strains, based on their antibiotic profiles. Strains of the Q-group produce the antibiotic gliotoxin and are generally considered to be more effective biocontrol agents [17]. The spectrum of secondary metabolites that is produced has a profound effect on the plant protection potential and mycoparasitic activity of *T. virens* [18,19]. Previous studies on Sm1 were carried out in strain Gv29-8, which is a Q-strain [19]. In order to relate our experiments on the role of Sm2 (and Sm1) in the interaction of *T. virens* with plants (see below) more directly to previous studies [5,13], we were interested whether strain I10, which we used for our studies [12], is a P or a Q-strain. *T. virens sm1* – but not *sm2* – knockout strains have been previously studied in strain Gv29-8. Both strains, I10 and Gv29-8, were grown for 36 h in shake flask cultivations in liquid potato dextrose broth medium and gliotoxin production was measured from filtered culture supernatants. The results (Additional file 1: Figure S4) showed that I10 produced 18.5 mg/l gliotoxin, an amount similar to strain Gv29-8 (17.5 mg/l), and strain I10 can therefore also be attributed to the group of Q-strains of *T. virens,* which is of particular relevance for the discussion of our plant experiments (see below).

### *Sm1* and *sm2* knockout strains show reduced levels of plant protection

**Figure 4:**
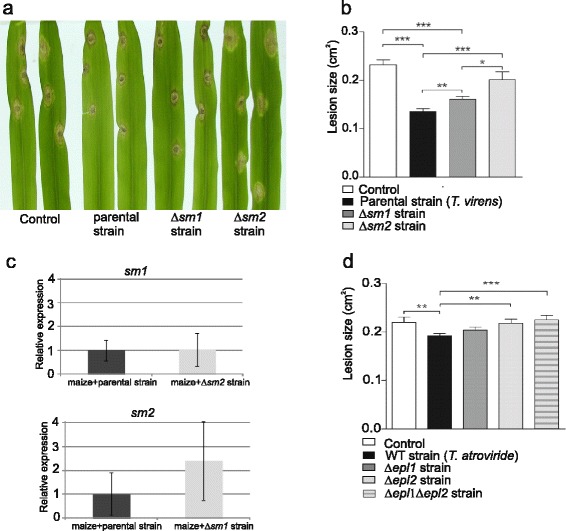
**Effect of** ***T. virens*** **(parental strain, ∆** ***sm1*** **and ∆** ***sm2*** **) on plant protection in maize seedlings challenged with the maize pathogen** ***C. heterostrophus*** **. (a)** Lesion development in leaves of *T. virens*-induced maize, two days after pathogen challenge. **(b)** Lesion size measured from photographed leaves. Number of lesions measured in control plant, 33; *T. virens* parental strain, 23; ∆*sm1*, 24; ∆*sm2*, 23. **(c)** Gene expression (qPCR) of *sm1* and *sm2* in maize seedlings infected with the pathogen *C. heterostrophus* and treated with the *T. virens* parental strain, *sm1* and *sm2* knockout strain. Number of lesions measured in control plant, 24; *T. atroviride* wild-type, 27; ∆*epl1*, 23; ∆*epl2*, 23; ∆*epl1*∆*epl2*, 24. Data from (b) and (d) represent the combined data from plants taken from two beakers. The experiment was carried out independently twice, with two biological repeats, each one containing 12 plants, with the same results within the variability. Bars indicate the SEM (standard error of the mean). *, ** and *** indicate significance at P < 0.05, 0.01 and 0.001. **(d)** Effect of *T. atroviride* (wild-type, ∆*epl1*, ∆*epl2*, and ∆*epl1*∆*epl2*) on plant protection in maize seedlings challenged with the maize pathogen *C. heterostrophus*. Lesion size measured from photographed leaves.

In order to test whether Sm2 is involved in the interaction of *Trichoderma* with plants we analyzed whether the lack of *sm2* leads to an altered potential to protect plants against fungal pathogens. For this, the interaction of *C. heterostrophus* with its host, maize, was used as a model pathosystem. We have recently standardized this system (N.L.L. and B.A.H., unpublished results) and it was used previously with *Trichoderma asperellum* ([20]). The *T. virens* parental and *sm1* knockout strains were also included, and in addition experiments were performed with *T. atroviride* wild-type, *epl1* and *epl2* knockout strains generated in a previous study [8]. Lesion sizes on maize plants, whose roots were co-cultured with the *T. virens* parental strain Δ*tku70* or *sm1* and *sm2* knockout strains, were measured (Figure 4a, b). Colonization of maize roots by the *T. virens* parental strain significantly decreased symptoms by more than 40%. Plants colonized by ∆*sm1* showed impaired resistance in comparison to plants treated with the *T. virens* parental strain, but were still significantly different from control plants, showing about 30% decrease in lesion size. Knock-out of *sm2*, however, led to a much more dramatic decrease in the ability of the fungus to induce resistance in maize, resulting in large lesions which were similar to the lesions of the control plants (no *Trichoderma*). In order to assess the gene expression of *sm*-genes in *T. virens* during plant interaction, root biomass was harvested from plant experiments four days post inoculation with *T. virens* conidia and analyzed by qPCR. Loss of one *sm*-gene might affect the expression of the other through feedback in the signaling network. The results (Figure 4c) showed, overall, no strong changes in gene expression of the other *sm*-gene. The expression of *sm1* was not altered in the Δ*sm2* strain. *sm2* expression tended to be higher in Δ*sm1* than in the parental strain, but this was not statistically significant.

Plant experiments were also carried out with *T. atroviride epl1* and *epl2* single and double knockout strains that were generated in a previous study [8]. While plants treated with the *T. virens* parental strain showed more than 40% reduction in lesion size, the lesion size in plants treated with the *T. atroviride* IMI206040 wild-type strain was only 13% smaller than in the control plants (Figure 4d). Despite the relatively poor plant protection potential of *T. atroviride*, we observed exactly the same trend regarding ability of ∆*epl1* and ∆*epl2* strains to protect plants as for the corresponding mutants in *T. virens.* In addition, lesion sizes on plants colonized by the double knockout strain ∆*epl1*∆*epl2* were not significantly different from ∆*epl2* treated plants and control plants (Figure 4d).

## Discussion

In this study the gene expression of the three genes encoding CPPs in *T. virens* was analyzed. In analogy to previous results from *T. atroviride*, *sm1* was found to be expressed during hyphal growth, i.e. under growth conditions when sufficient nutrients are available and fast biomass formation occurs. For *sm2* gene expression was detected in mycelium that was harvested from sporulating cultures. Since the formation of conidia is associated with differentiation of the hyphae to form conidiophores and phialides, *sm2* gene expression could also be associated with these structures, but it is unfortunately not possible to separate them efficiently. It should be noted that in shake flask cultivations, where, according to our microscopic observations, large amounts of chlamydospores - which directly split off from hyphae - but no conidia, were formed, no expression of *sm2* was found (Figure 1 and Figure S2 in supplemental material). This indicates that *sm2* gene expression is connected to the formation and maturation of conidia but not to other types of spores in *T. virens*. It should be noted that, due to morphological differences of the mycelium on agar plates between *T. atroviride* and *T. virens*, in *T. atroviride* the harvested biomass from sporulating cultures consists mainly of spores, whereas in *T. virens* I10 the mycelium is fluffier and the harvested biomass is therefore a mixture of spores and hyphae. This probably explains why in *T. virens* expression of *sm1* and *sm2* was detected in these samples, whereas in *T. atroviride* strong *epl2* expression but only weak *epl1* expression was found. *Sm2* is also expressed in co-culture with maize (Figure 4).

Our transcriptional data for *sm1* are in agreement with findings by Djonovic et al. [5], who reported expression under all tested conditions, including sporulating and non-sporulating mycelia. In other fungi there is also evidence that homologues of *sm1* are expressed during hyphal growth, e.g. in *B. cinerea*, *bcspl1* was found to be expressed under many different growth conditions, whereas no expression was found for *bcspl2*, a second CP gene [21]. *MgSM1* from *Magnaporthe grisea* was also expressed during different fungal growth stages [22]. In addition to these gene expression data, the protein Epl1 was found to be the predominant protein in the secretome of submerged *T. atroviride* cultivations with glucose as a carbon source [6]. In the plant pathogenic basidiomycete *Moniliophthora perniciosa* gene expression data of the 12 CP genes (*MpCP1-12*) showed complex transcriptional profiles throughout fungal development and pathogenic infestation of the plant, suggesting a specialization of the respective proteins in different biological processes [23]. In ascomycetes, gene expression data are so far limited to homologues of *sm1* except for *epl2* and *epl3* from *T. atroviride* and *bcspl2* from *B. cinerea* (see above). It will be of interest for future studies to obtain more expression data for these genes, in particular considering the strong effect of the lack of *sm2* on the ability of *Trichoderma* to protect maize from *C. heterostrophus* in our plant experiments (Figure 4).

The mycoparasitic potential of *sm*-knockout strains against *R. solani* and *B. cinerea* was not altered and it can be anticipated that this would also be the case for other host fungi. Nonetheless *sm*-knockout strains have a strong effect on the biocontrol properties of *T. virens* via direct effects on the *Trichoderma*-plant interaction. In the *T. virens* Gv29.8-maize - *Colletotrichum graminicola* interaction, loss of Sm1 resulted in complete loss of the capacity to reduce lesion size on the leaves [13]. A single protein might seem unlikely to be responsible, alone, for the induction of systemic resistance. However, it seems that in the maize *C. graminicola* interaction Sm1 is indeed the dominant player. In the maize - *Cochliobolus* assay used in this study, on the other hand, knock-out of *sm1* reduced the plant protection potential of *T. virens*, but in this pathosystem the lack of Sm2 had an even greater effect and lesion size of maize leaves was statistically not different from the control (no *Trichoderma*). Although the colonization efficiency of maize roots by *T. virens* was not directly measured, it should be noted that upon harvesting of plant roots for biomass extraction for qPCR experiments, no obvious phenotypes in fungal growth and the appearance of the colonized roots were observed. In plant experiments with *T. atroviride* plant protection levels were overall lower than with *T. virens*, but the same trend was observed for *epl1* and *epl2* knockout strains as for *sm1* and *sm2* knockout strains, confirming that Sm2/Epl2 are, in the *C. heterostrophus*-maize pathosystem, more important for plant protection than Sm1/Epl1. This is also relevant because it underlines that the observed effect is not due to any unwanted, genetic side-effects of the knockout strains or due a particular feature of the *T. virens* strain used. The *T. atroviride* double knockout strain Δ*epl1*Δ*epl2* appeared slightly (albeit not significantly) less effective than the Δ*epl2* strain (Figure 4b, d). These data are compatible with an additive contribution of Sm1 and Sm2, and the contribution of these two paralogs to the induction of resistance is similar for *T. virens* and *T. atroviride*. The data in Figure 4 provide genetic evidence that for maximal induction of resistance, both paralogs need to be present. In contrast to maize- *C. graminicola*, where Sm1 dominates, Sm2 appears to be the dominant one in this particular assay. Since *sm1* expression was not found to be altered in the *sm2* deletion strain in plant experiments it can be concluded that reduction of the capacity to protect the plant was directly due to the absence of Sm2.

When comparing the *C. graminicola* to the *C. heterostrophus* pathosystem assays, it is important to note that the assay here was done using hydroponic cultures rather than soil-grown plants, which could affect the relative extent of colonization, intensity of ISR and contribution of specific secreted proteins. To maximize the potential of *Trichoderma* spp. to protect plants, expression of different combinations of CPP family members, at different levels, will need to be tested, in different pathosystems.

## Conclusions

CPPs are potent inducers of plant defense responses in plant pathogenic fungi as well as plant-beneficial fungi such as *Trichoderma* species. In this study we showed that *T. virens sm2* knockout strains were more impaired in the protection of maize seedlings against the pathogen *C. heterostrophus* than *sm1* knockout strains. *T. atroviride* was overall less effective in plant protection than *T. virens*, but the same trend was observed for the respective *epl2* and *epl1* knockout strains. These findings advance our understanding of the diversified functions of CPPs in fungi and of the pool of molecules that are involved in the beneficial interaction of *Trichoderma* with plants. Our results show that the paradigm of Sm1 as the main or exclusive inducer of plant systemic resistance triggered by *Trichoderma*-root interactions needs to be generalized. As we have shown that in the particular interaction studied here Sm2 is even more important than Sm1, it seems likely that even more elicitors remain to be discovered in *Trichoderma*.

## Methods

### Generation of knockout strains and phenotype analysis

Knockout strains were generated using *T. virens* I10 Δ*tku70* [12] as a parental strain. A schematic representation of the *sm1* and *sm2* knockout loci is shown in Additional file 1: Figure S1. All primers used for generation and verification of knockout strains are listed (see Additional file 1: Table S1). For construction of the *sm1* deletion vector 900 bp of the 5’- and 860 bp of the 3’-flanking regions of *sm1* were amplified from genomic DNA of *T. virens* I10 with the primers sm1-5’-fw/sm1-5’-rv and sm1-3’-fw/sm1-3’-rv, respectively. The PCR products were cloned into a pBS (Bluescript SK+) vector containing a selection hph-cassette (*hph* gene, conferring resistance to hygromycin, under control of the *T. reesei pki* promoter and *cbh2* terminator) [24]. The obtained plasmid was first linearized with XhoI and the *sm1*-5’flanking region was inserted with the In-Fusion cloning kit (Clontech, Mountain View, CA, USA). Then the resulting plasmid was again linearized with EcoRV and the *sm1*-3’ flanking region was inserted. For identification of knockout strains primers sm1-promupstream-fw and sm1-hph-cass-rv were used, yielding a 2 kb PCR product for positive knockout strains. The purification of the knockout strains and thus absence of the *sm1* gene was verified with primers sm1-promupstream-fw and sm1-term-rv, yielding a PCR product with the size of 2 kb for the *sm1*-wild-type.

For generation of the *sm2* deletion vector the *Aspergillus oryzae ptrA* gene was used as a selection marker, conferring resistance against pyrithiamine [25]. The resistance marker cassette, containing the native promoter and terminator of the *ptr* gene, was amplified from a plasmid (kindly obtained from B. Seiboth) and inserted into a pBS (Bluescript SK+) vector that was previously linearized with XhoI and HindIII via In-Fusion cloning (Clontech). The 5’-flanking region of *sm2* was amplified with the primers sm2-5’-fw/sm1-5’-rv and the 3’-flanking region was amplified with the primers sm2-3’-fw/sm2-3’-rv. The flanking regions were inserted into plasmid pBS-ptr that was linearized with XhoI for the s*m2*-5’ region and HindIII for the *sm2*-3’ region via In-Fusion cloning (Clontech). Knockout strains were identified by PCR with the primers sm2-promupstream-fw and sm2-ptr-cass-rv, yielding a 2 kb band for the *sm2* knockout locus. After single spore isolations, the absence of the *sm2* gene was verified with the primers sm2-promupstream-fw and sm2-term-rv, yielding a 2.2 kb band for the *sm2* wild-type locus.

Fungal transformation, carried out with the PCR-amplified transformation cassettes, protoplast generation, preparation of selection media and purification of fungal transformants were performed as described in [12]. For selection on pyrithiamine (1 μg/ml) *T. virens* transformants were grown on ISM medium; 0.68 g/L KH_2_PO_4_, 0.87 g/L K_2_HPO_4_, 1.7 g/L (NH_4_)_2_SO_4_, 0.2 g/L CaCl_2_, 0.2 g/L KCl, 0.2 g/L MgSO_4_.7H_2_O, 5 mg/L FeSO_4_.7H_2_O, 2 mg/L ZnSO_4_.7H_2_O, 2 mg/L MnSO_4_.7H_2_O [6] in order to facilitate the differentiation between transformants and background growth.

Mycoparasitism assays were performed on potato dextrose agar (PDA) plates. *T. virens* and a host fungus (*R. solani* or *B. cinerea*) were placed on opposite sides of the agar plate and incubated at 28°C with a 12 h/12 h light/dark cycle. Images of the confrontation assays were taken every 24 h to record the antagonism and overgrowth of the host fungi by *T. virens*.

Phenotype analysis of the knockout strains was carried out as described for *T. atroviride* in [8]. All experiments were carried out with at least two independent biological replicates.

### Fungal cultivations and gene expression analysis

Shake flask cultivations were carried out with ISM medium [6] containing 1% glucose and 0.05% peptone. Media were inoculated with 1 × 10^6^ conidia/ml and cultivated at 25°C and 200 rpm. Mycelia were harvested at the time points indicated in the results section and frozen in liquid nitrogen. For gene expression analysis from conidia at different maturation stages (based on the appearance of the mycelium covered with conidia, ranging from white to first light green and then dark green conidia), sporulated mycelia were scraped from PDA plates with a spatula and frozen in liquid nitrogen. For RNA isolation the samples were ground to a fine powder under liquid nitrogen and total RNA was isolated using the guanidinium thiocyanate method [26]. Isolated RNAs were treated with DNAse I (Fermentas, St Leon-Rot, Germany), and cDNAs were generated with the Revert Aid H-minus cDNA synthesis kit (Fermentas). RT–PCR (25 cycles) was performed using the gene-specific primers listed (see Additional file 1: Table S1). Accession numbers of the *sm*-genes in the JGI *T. virens* genome database; http://genome.jgi-psf.org/TriviGv29_8_2/TriviGv29_8_2.home.html) are: *sm1* 110852, *sm2* 111830 and *sm3* 32154. The corresponding accession numbers in the NCBI database are EHK25601 for Sm1, EHK20677 for Sm2, and EHK25819 for Sm3. The *tef1* gene (translation elongation factor 1 alpha, protein ID 83874 in the JGI database and in the NCBI database EHK22702) was used as reference gene.

qPCR reactions were performed in an Eppendorf Realplex thermal cycler. The reaction mix contained 12.5 μl SYBR green Supermix (Bio-Rad Laboratories, Hercules, CA, USA), 8.5 μl pure water, 6.25 μM forward and 6.25 μM reverse primer, and 2 μl 1:50 diluted template cDNA (5 μg of RNA/reaction were reverse-transcribed using the Revert Aid H-minus cDNA synthesis kit (Fermentas)). Reactions were performed in triplicates. Primer efficiency was calculated using a dilution series from 1:5 to 1:5000 with the PCR baseline-subtracted mode. The amplification protocol consisted of an initial denaturation step for 3 min at 95°C followed by 40 cycles of denaturation (95°C for 15 s), annealing, and elongation (60°C for 15 s). Oligonucleotides are listed in Additional file 1: Table S1. The *tef1* gene was used as a reference. Expression data were evaluated using REST software [27]. Cultivations for gene expression analysis were carried out with at least two independent biological replicates.

### Gliotoxin measurements

LC-MS/MS screening of fungal metabolites was performed with a QTrap 5500 LC-MS/MS System (Applied Biosystems, Foster City, CA) equipped with a TurboIonSpray electrospray ionization (ESI) source and a 1290 Series HPLC System (Agilent, Waldbronn, Germany). Chromatographic separation was performed at 25°C on a Gemini® C_18_-column, 150 × 4.6 mm i.d., 5 μm particle size, equipped with a C_18_ 4 × 3 mm i.d. security guard cartridge (all from Phenomenex, Torrance, CA, US). The chromatographic method, as well as chromatographic and mass spectrometric parameters for 186 of the investigated analytes, is as described by Vishwanath et al. [28]. In the meantime, the method has been further expanded to cover 320 metabolites.

ESI-MS/MS was performed in the time-scheduled multiple reaction monitoring (MRM) mode both in positive and negative polarities in two separate chromatographic runs per sample by scanning two fragmentation reactions per analyte. The MRM detection window of each analyte was set to its expected retention time ± 27 seconds and ± 48 seconds in the positive and the negative mode, respectively. The target cycle time was 1 second. Confirmation of positive analyte identification was obtained by the acquisition of two MRMs per analyte (with the exception of moniliformin and 3-nitropropionic acid, that exhibit only one fragment ion), which yielded 4.0 identification points according to commission decision 2002/657/EC. In addition, the LC retention time and the intensity ratio of the two MRM transition agreed with the related values of an authentic standard within 0.1 min and 30% rel., respectively.

For further confirmation of the identity of gliotoxin, Enhanced Product Ion scans were performed using the third quadrupole as linear ion trap, applying a collision energy of 35 V, a collision energy spread of 15 V and a dynamic fill time of the trap. Spectra were obtained by averaging 20 scans of a scan speed of 1000 amu/sec and a scan range of 50–820 amu.

### Plant assays for induced systemic resistance

We used a hydroponic system [5,29] to evaluate the resistance response of maize seedlings stimulated by *T. virens* or *T. atroviride*. 600 ml glass beakers were filled with 200 ml plant nutrient solution (half-strength Murashige and Skoog basal medium, 2.5 mM MES buffer pH = 5.7). A perforated stand for supporting the seeds was made from a 200 μl tip holder. Maize seeds (Royalty, local hybrid, purchased from Ben Shachar, Tel Aviv) were surface sterilized by dipping them in 10% H_2_O_2_ for three hours, followed by three washes with sterile water. Treated seeds were dried on sterile Whatman #1 paper, placed in sterile Petri dishes containing half-strength Murashige and Skoog agar and incubated in the dark for three days at 30°C to allow germination. 12 germinated seeds with similar-sized roots and shoots were placed on the stands in each aseptic beaker. The plants were maintained in a controlled environment at 23°C and a 16 h photoperiod with moderate shaking on an orbital shaker (100 rpm). After four days of growth in the beakers, plants were inoculated with *Trichoderma* spore suspension to a final concentration of 5×10^3^ spores/ml. Roots and fungus were allowed to interact for four more days before pathogen challenge. For pathogen challenge, plants - with their roots - were taken out of the beakers and the second leaf of each plant was attached to a tray from the edges of the leaf. The roots of each set of plants according to treatment were wrapped separately in wet paper towels.

The maize pathogen *C. heterostrophus* (strain C4) was grown for seven days on complete xylose medium [30] in the same controlled environment as the plants. The second leaf was inoculated with 7 μl droplets of 0.02% Tween 20 in double distilled water containing 1000 spores. Trays were closed in clear plastic bags to keep the plants moist and kept in the controlled environment. Pictures of the challenged leaves were taken after 48 h and lesions were measured using ImageJ software (http://imagej.nih.gov/ij/). For each treatment the data represent at least eight leaves, each with three lesions from two biological repeats.

For analysis of gene expression, roots with adhering *Trichoderma* were harvested from hydroponic cultures grown in parallel to those used for the ISR assays. The roots were washed gently with culture medium, frozen and ground to a fine powder in liquid nitrogen. RNA was extracted using Tri Reagent (MBC Molecular Research Center, Cincinnati, OH, USA) following the manufacturer’s protocol, and cDNA was synthesized as described above.

### Supporting data

The data sets supporting the results of this article are included within the article and its additional files.

## Additional file

Additional file 1:
**Figure S1.** Generation of *sm1* and *sm2* knockout strains. **Figure S2.** Chlamydospore formation in *T. virens* shake flask cultivations. **Figure S3.** Mycoparasitism confrontation assays of *T. virens* against *R. solani*. **Figure S4.** Gliotoxin measurements of *T. virens* strains I10 and Gv29-8. **Table S1.** Primers used in this study.

## Abbreviations

CPP: Cerato platanin protein
CP: Cerato platanin
PDA: Potato dextrose agar
