# Supplementary material for: Sm2, a paralog of the Trichoderma cerato-platanin elicitor Sm1, is also highly important for plant protection conferred by the fungal-root interaction of Trichoderma with maize
Source: BMC Microbiol. 2015 Jan 16;15(1):2. doi: 10.1186/s12866-014-0333-0 (PMC4320488; doi:10.1186/s12866-014-0333-0)
Supplement: Additional file 1: — Figure S1. Generation of sm1 and sm2 knockout strains. Figure S2. Chlamydospore formation in T. virens shake flask cultivations. Figure S3. Mycoparasitism confrontation assays of T. virens against R. solani. Figure S4. Gliotoxin measurements of T. virens strains I10 and Gv29-8. Table S1. Primers used in this study. [file 12866_2014_333_MOESM1_ESM.pdf]

Fig. S1

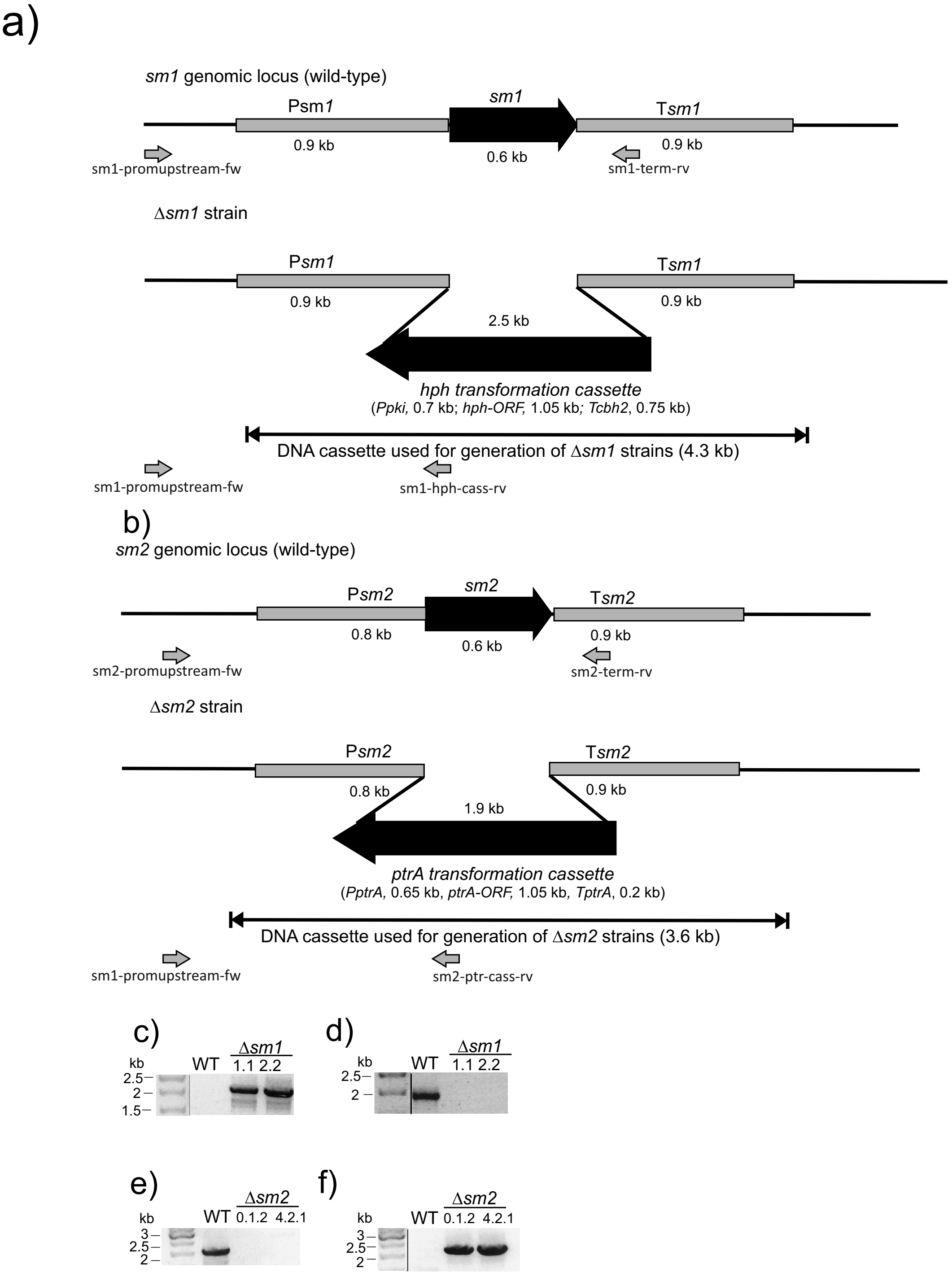

FIG. S1. Generation of *sm1* and *sm2* knockout strains. Genomic locus maps for the generation of (a) Δ*sm1* and (b) Δ*sm2*.

(a) In the parental strain (Δ*tku70* strain) the *sm1* gene was replaced with the *hph*-gene from *Escherichia coli* (using the *Trichoderma reesei pki* promoter and *cbh2* terminator), enabling selection of transformants on medium containing hygromycin. (b) In the parental strain (Δ*tku70* strain) the *sm2* gene was replaced with the *ptrA*-gene from *Aspergillus oryzae* (native promoter and terminator), enabling selection of transformants on medium containing pyrithiamine. (c-f) Identification and verification of *sm1* and *sm2* knockout strains. The positions of the primers used for identification of the knockout strains are indicated with arrows and the respective primer names. Primer sequences are listed in Supplementary Table 1.

(c) Identification of *sm1* knockout strains, yielding a 2.0 kb PCR product for knockout strains and (d) absence of the *sm1* wild-type locus, using the primers listed in Supplementary Table 1. Two positive *sm1* knockout strains (1.2 and 2.2 are shown). Strain 1.2 was used for further experiments. (e) Identification of *sm2* knockout strains, yielding a 2.0 kb band for the knockout locus and (f) absence of the *sm2* gene using the primers listed in Supplementary Table 1. Two positive *sm2* knockout strains (0.1.2 and 4.2.1 are shown). Strain 4.2.1 was used for further experiments.

Fig. S2

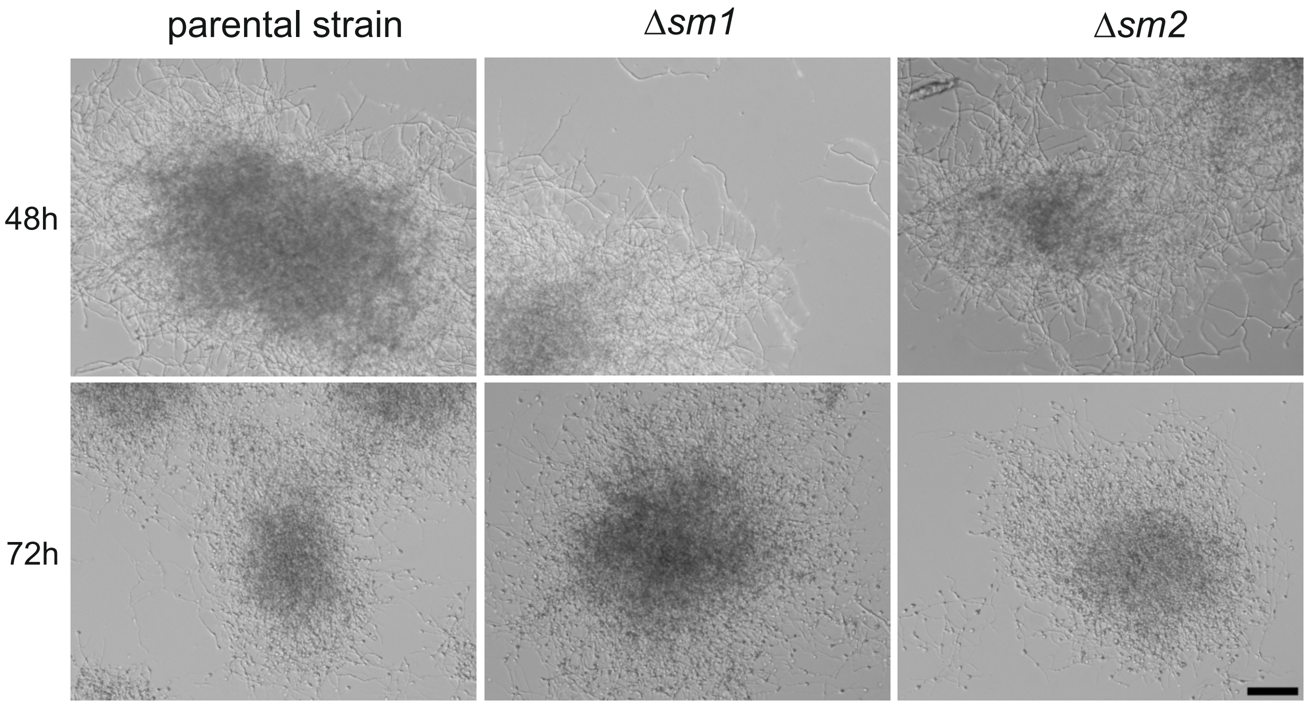

FIG. S2. Chlamydospore formation in *T. virens* shake flask cultivations. Microscopic analysis of hyphal growth and chlamydospore formation of the *T. virens* parental strain, Δ*sm1* and Δ*sm2* knockout strains at the time points 48 h and 72 h. Scale bars = 100µm.

Fig. S3

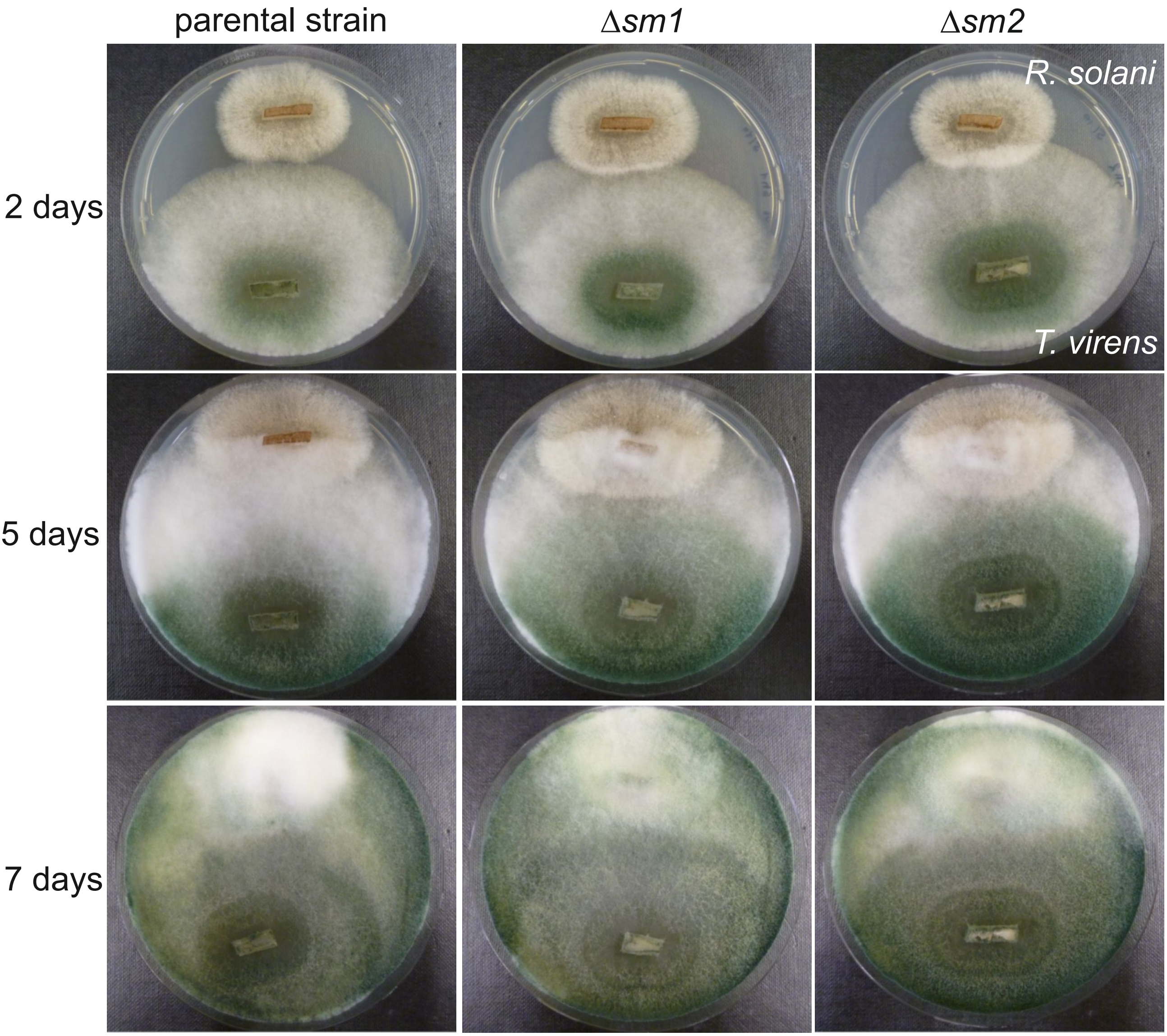

FIG. S3. Mycoparasitism confrontation assays of *T. virens* against *R. solani*.

FIG. S4

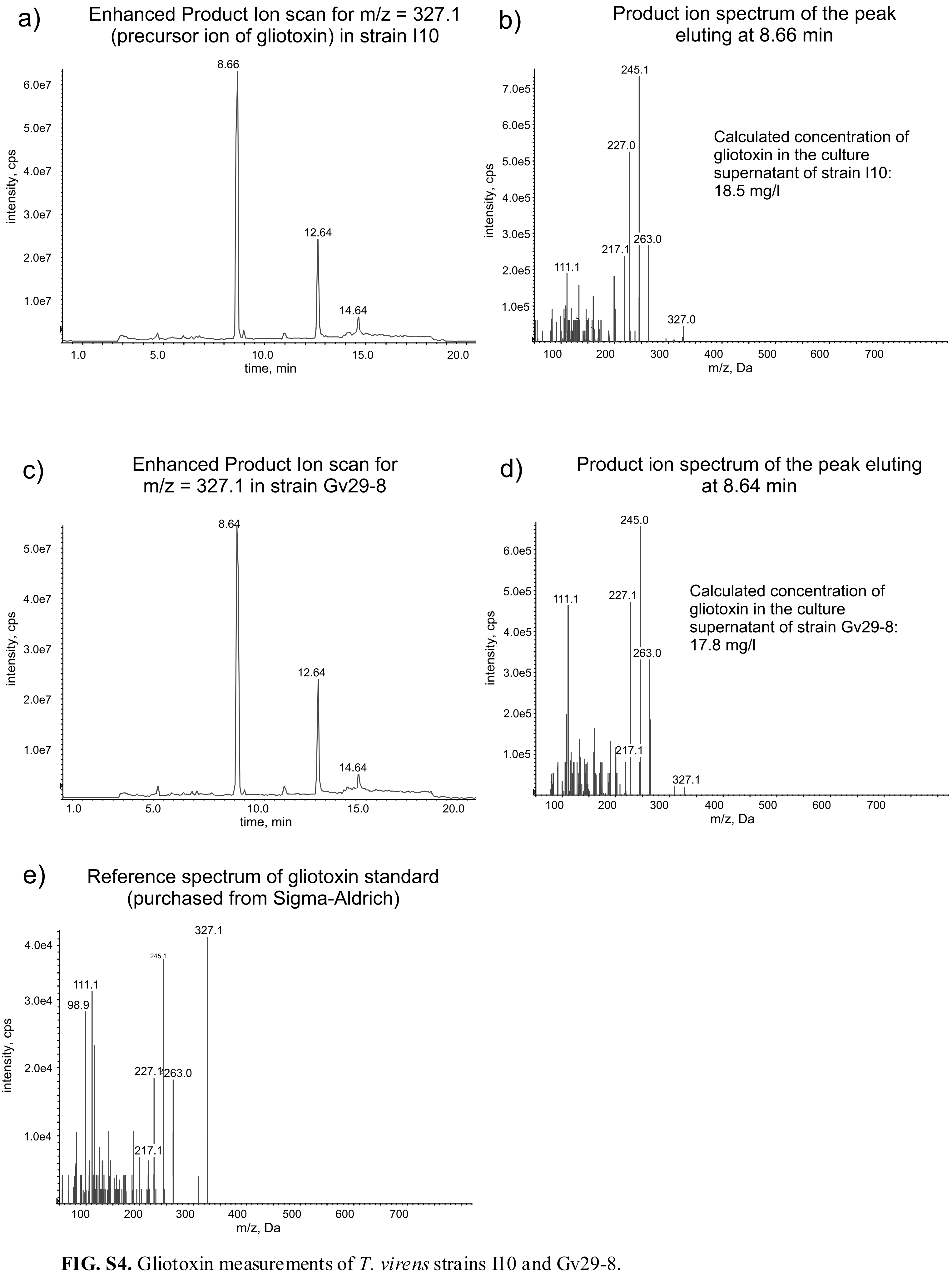

FIG. S4. Gliotoxin measurements of *T. virens* strains I10 and Gv29-8.

SUPPLEMENTARY TABLE 1. Primers used in this study

**Primers for *sm1* and *sm2* knockout strains**	
sm1-5'-fw	GTACCGGGCCCTCGAGTGAATTGAATGGATTGACTG
sm1-5'-rv	ACGAACGGTACTCGAGGCTTAACTGCGAGAATGG
sm1-3'-fw	ACGGTAAAGCTTGATATCCATCTTCAAAGAAGTTCG
sm1-3'-rv	CTGCAGGAATTCGATATCAGCCGCTTACTTCTTCC
sm1-promupstream-fw	GAGAGAGTTCCATGGCG
sm1-term-rv	ATTCACGCCTGGCACAGA
sm1-hph-cass-rv	TAGAAGTACTCGCCGATAGT
sm2-5'-fw	GTACCGGGCCCTCGAGCTGCGGCTACACATA
sm2-5'-rv	ACGAACGGTACTCGAGATGGAGGCGAGTTAG
sm2-3'-fw	ACGAACGGTAAAGCTTAATAGGAAAATGACGTG
sm2-3'-rv	ATTCGATATCAAGCTTCAGACGATTATAAGCATGA
sm2-promupstream-fw	ATCATACTTGCCAAAGCGAC
sm2-term-rv	GTGATGTGGGAGTAGTGG
sm2-ptr-cass-rv	CCACTTGCCACCGAAATG
**RT-PCR primers**	
tef1 fw	GTCGTTACCTTCGCTCCTTCCAA
tef1 rv	CGGACTTGATGAACTTGGGGGC
sm1 fw	CCAACATCTTCACTCTCGCTCTC
sm1 rv	AATGTTGAAGCCAGAAGCAGCGTG
sm2 fw	CCTCTTCAATGCTGCTACCCTC
sm2 rv	GCCATCAGTAAGAGTGTCCAGTG
sm3 fw	GCTGCCATTGTCGCCCCG
sm3 rv	CACCAATGAACCCAACTGCGTC
**qPCR primers**	
tef1 qPCR fw	CCACATTGCCTGCAAGTTCGC
tef1 qPCR rv	GTCGGTGAAAGCCTCAACGCAC
qPCR-SM1-fw sm1 rv	GCTCGATGCCATGAATGCTCTG
qPCR-SM1-rv sm2 rv	TGAGTAGCGGTAGCAGAAACGC
qPCR-SM2-fw sm3 rv	CCTGCTCTGACGGTGTCAAT
qPCR-SM2-rv	GATACCTTGGACGCCACCAA
